# Absence of Birth-Weight Lowering Effect of ADCY5 and Near CCNL, but Association of Impaired Glucose-Insulin Homeostasis with ADCY5 in Asian Indians

**DOI:** 10.1371/journal.pone.0021331

**Published:** 2011-06-21

**Authors:** Senthil K. Vasan, Matt J. Neville, Belavendra Antonisamy, Prasanna Samuel, Caroline H. Fall, Finney S. Geethanjali, Nihal Thomas, Palany Raghupathy, Kerstin Brismar, Fredrik Karpe

**Affiliations:** 1 Department of Molecular Medicine & Surgery, Karolinska Institutet, Stockholm, Sweden; 2 Department of Endocrinology, Diabetes & Metabolism, Christian Medical College, Vellore, India; 3 Oxford Centre for Diabetes, Endocrinology and Metabolism, University of Oxford, Oxford, United Kingdom; 4 Department of Biostatistics, Christian Medical College, Vellore, Tamil Nadu, India; 5 Medical Research Council (MRC) Epidemiology Resource Centre, Southampton, United Kingdom; 6 Department of Clinical Biochemistry, Christian Medical College, Vellore, Tamil Nadu, India; 7 Department of Child Health, Christian Medical College, Vellore, Tami Nadu, India; 8 National Institute for Health Research (NIHR) Oxford Biomedical Research Centre, Oxford Radcliffe Hospitals (ORH) Trust, Oxford, United Kingdom; University Institute of Social and Preventive Medicine, Switzerland

## Abstract

**Background:**

A feature of the Asian Indian phenotype is low birth weight with increased adult type 2 diabetes risk. Most populations show consistent associations between low birth weight and adult type 2 diabetes. Recently, two birth weight-lowering loci on chromosome 3 (near *CCNL1* and *ADCY5*) were identified in a genome-wide association study, the latter of which is also a type 2 diabetes locus. We therefore tested the impact of these genetic variants on birth weight and adult glucose/insulin homeostasis in a large Indian birth cohort.

**Methodology/Principal Findings:**

Adults (n = 2,151) enrolled in a birth cohort (established 1969-73) were genotyped for rs900400 (near *CCNL1*) and rs9883204 (*ADCY5*). Associations were tested for birth weight, anthropometry from infancy to adulthood, and type 2 diabetes related glycemic traits. The average birth weight in this population was 2.79±0.47 kg and was not associated with genetic variation in *CCNL1* (*p* = 0.87) or *ADCY5* (*p* = 0.54). Allele frequencies for the ‘birth weight-lowering’ variants were similar compared with Western populations. There were no significant associations with growth or adult weight. However, the ‘birth weight-lowering’ variant of *ADCY5* was associated with modest increase in fasting glucose (β 0.041, *p* = 0.027), 2-hours glucose (β 0.127, *p* = 0.019), and reduced insulinogenic index (β -0.106, *p* = 0.050) and 2-hour insulin (β -0.058, p = 0.010).

**Conclusions:**

The low birth weight in Asian Indians is not even partly explained by genetic variants near *CCNL1* and *ADCY5* which implies that non-genetic factors may predominate. However, the ‘birth-weight-lowering’ variant of *ADCY5* was associated with elevated glucose and decreased insulin response in early adulthood which argues for a common genetic cause of low birth weight and risk of type 2 diabetes.

## Introduction

A recent genome-wide association (GWA) study identified two independent loci on chromosome 3 (rs900400 near *CCNL1* and rs9883204 of *ADCY5*) for low birth weight [1]. When replicated in Europeans robust associations (*p* = 3×10^−26^ and 3×10^−9^, respectively) with birth weight were observed leading to a 113 g difference between homozygous carriers vs. non-carriers for the two respective variants [1].

Indian new born babies generally weigh the least in the world with a mean birth weight at term being 2.6 to 2.9 kg compared to 3.5 to 3.7 kg among Europeans [2], [3]. The discovery of genetic variants having a strong effect on birth weight therefore provides the opportunity to test if some of the low Indian birth weight has a genetic component.

A systematic review of the relationship between birth weight and subsequent risk of development of type 2 diabetes in adulthood has shown an inverse relationship that is consistent between populations [4]. Although the exact mechanism for this relationship remains unclear, the fetal-insulin hypothesis describes a distinct role for a genetic and environmental component for the association [5]. An alternative, but neither opposing, nor exclusive, explanation is through fetal programming due to the *in utero* environment [6]. However, this latter explanation remains mechanistically elusive.

Genetic variants that influence fetal insulin secretion or insulin sensitivity appear to be important determinants of fetal growth, and subsequent development of type 2 diabetes [7]. Associations between mutations in the *INS, INSR, IPF1, KCNJ11, ABCC8* and *HNF1B* genes reducing fetal insulin secretion and markedly reducing birth weight in monogenic diabetes provide support for a genetic role in the modulation of birth weight, although this cannot be extrapolated to the general population due to the rarity of these mutations [5], [8]–[10]. There is some evidence from the study of common genetic variants related to type 2 diabetes (*TCF7L*, *GCK,* I/III polymorphisms of *INS*-VNTR and *IGF-1*) and its effects on birth weight, lending support to the notion that more moderate impairment of insulin secretion provides a link between inheritance of type 2 diabetes susceptibility and reduced weight at birth [11]–[14]. More recently genetic variants near *ADCY5* have been linked to glycemic traits in healthy individuals [15], and with birth weight in the Danish Inter99 population [16].

Therefore, we aimed to replicate two ‘birth weight-lowering’ alleles (rs900400 near *CCNL1* and rs9883204 of *ADCY5*) in a longitudinal birth cohort form South India and also test whether the low Indian birth weight is driven, or partly explained, by similar genetic factors as in Europeans. In this replication there are two inherent questions. First, whether the low birth in Indians is due to higher frequencies of ‘birth weight-lowering’ alleles compared to the Western population and secondly, whether the lower average birth weight is modulated by variants in *ADCY5* and near *CCNL1* in a way similar to the western population. Due to the known associations between birth weight and adult type 2 diabetes and the fact that one of the ‘birth weight-lowering’ alleles (*ADCY5*) also appears to be a genetic determinant of glycemic traits, we also aimed to investigate possible associations of *CCNL1* and *ADCY5* genotypes with diabetes related intermediate-traits in addition to investigating the influence of these SNPs on anthropometric traits during longitudinal follow-up from birth to adulthood.

## Materials and Methods

### Ethics Statement

All study participants gave written informed consent and the protocol was in accordance with the Helsinki Declaration, and approved by the Institutional Ethics committee and Review board of Christian Medical College & Hospital, Vellore, India.

### Cohort description

The current study included 2,151 adults aged 26–32 (mean 28.3±1.10) years, drawn from an original birth cohort of 10,691 singleton births during 1969–1973 in one of 24 wards of Vellore town and adjoining rural villages, Tamil Nadu, India. Details of the original cohort are described elsewhere [17]. Complete birth measurements were available for 4,092 (2,790 from rural and 1,302 from Urban dwelling) individuals and 2,218 out of 2,572 members retraced during 1999–2002 agreed to take part in a study of glucose tolerance and cardiovascular risk factors, reported earlier [18]. The original cohort was followed up at several time points of growth and development from birth, through infancy (1–3 months), childhood (6–8 years) and adolescence (10–15 years) and adulthood. A total of 2,151 (1,175 of rural birth and 976 of urban birth) participants (out of 2,218 retraced) had adequate DNA samples and were included in the current study. Based on WHO criteria, there were 83 (3.9%) subjects with impaired fasting glucose (IFG) (>6.1 but below 7.0 mmol/l), 319 (14.8%) with impaired glucose tolerance (IGT) (2-hours blood glucose >8 mmol/l) and 55 (2.6%) with manifest type 2 diabetes (T2DM), demonstrated by an oral glucose tolerance test.

### Anthropometric measurements and biochemical analysis

Anthropometric measurements included height, weight, waist and hip circumference measured by standard methods during adulthood. Body fat percentage was calculated from four skinfold measurements [19], [20]. Plasma glucose and insulin were recorded at 0, 30, 60 and 120 minutes, following a 75 g oral glucose tolerance test (after 12 hours of overnight fasting). Plasma glucose concentrations were measured by glucose oxidase/peroxidase methods, and serum lipids using commercial enzymatic kits (Roche Diagnostics, Germany) on a Hitachi 911 autoanalyser (USA). Plasma insulin was measured by an immunoradiometric assay using Coat-a-Count kits (Diagnostic Products Corporation, USA). The quality of these measurements was assessed using Roche Precinorm and Precipath controls for glucose and lipids and BioRad Lyphocheck Immunoassay controls for insulin. Intra- and inter-assay coefficients of variation for insulin estimations were 8.0–14.5 and 8.2–13.0% respectively. HOMA-IR and HOMA-B were calculated using an algorithm from http://www.dtu.ox.ac.uk/homacalculator/index.php. All biochemical measurements were done centrally at the Department of Clinical Biochemistry, Christian Medical College & Hospital, Vellore, on samples that were collected during adulthood (in 2002).

### Genotyping

DNA was extracted from peripheral blood using Qiagen kits. The samples were genotyped using 10–20 ng genomic DNA in 384-well format on an ABI 7900 machine at final volume of 4 µl. The genotyping was performed using TaqMan® SNP Genotyping Assays C1860681_10 and C3035719_20 for rs900400 (near *CCNL1*) and rs9883204 (*ADCY5*), respectively. The TaqMan® genotyping master mix was used following the manufacturer's conditions. Genotyping quality control was tested in 8.6% of the samples (genotyped in duplicate) with 0% difference in genotype. Genotyping failed in 65 (3.0%) for rs900400 and 61 (2.8%) for rs9883204, most probably due to low quality DNA for the platform used.

### Statistical methods

We tested the association between quantitative phenotypes and SNPs using ANOVA for normally distributed variables and Kruskal–Wallis test for skewed variables, adjusted for gender. Linear regression analysis was performed for each of the glycemic trait as the dependent variable against genotypes (additive model) as independent variables, with age, gender, consanguinity and BMI as covariates. Z-scores for selected anthropometric variables were age-adjusted and converted into within-cohort age- and sex-specific Z-scores [(subject mean- cohort mean)/cohort SD] considering variations in ages measured at different time points. The cohort mean and SD were derived from all 4,092 individuals with complete birth and parental measurements. Infant data were included if there was at least one measurement between 1 and 3 months, and the latest available time point used. Childhood data were included if there was at least one measurement between 6 and 8 years, and the average Z-score was used if there were more than one measurement. Adolescent data were included if there was at least one measurement between 10 and 14 years, the Z-score for the age closest to 12 years being selected. The main analysis used all available data at each time point. The absolute effect of genetic variants on Z-scores of anthropometry was assessed using ANOVA after adjustment for gestational age, gender and consanguinity. Effect size of the additive model was computed using regression models adjusted for gestational age, gender and consanguinity. All data were analyzed using STATA (Version 11.0). Subjects with T2DM (n = 55) were excluded from all analysis. All diabetes related traits except 2 hours postprandial glucose and AUC glucose were log transformed to obtain normal distribution before analysis.

A power calculation based on variance explained by both the genetic variants (0.3% for *CCNL1* and 0.1% for *ADCY5*) as previously described, showed a power of 74% and 32% to detect a variance (*r* ^2^) explained, for *CCNL1* and *ADCY5* respectively using adjusted models, calculated at an alpha (0.05), and a sample size of 2,151. The coefficient of variation (CV) of birth weight was 18.8% for *CCNL1* and 16.7% for *ADCY5* and this was comparable to the effects demonstrated by these variants on birth weight as described by Freathy RM et al [1].

## Results

There were no significant associations between either rs900400 (near *CCNL1*) or rs9883204 (*ADCY5*) with birth weight in the Indian population (Table 1). The allele frequencies of the ‘birth weight-lowering’ alleles were 0.21 for C allele (rs900400) and 0.81 for C allele (rs9883204). The rs9883204 showed some loss of expected heterozygosity and did not follow Hardy–Weinberg equilibrium (X2 4.45, *p* = 0.035) in the cohort. This is a well-known phenomenon in cohorts with a high degree of consanguineous parentage, which was the case in 47% of the rural part of the cohort and 29% in the urban group.

**Table 1:** Demographic characteristics of the birth cohort.

	CCNL1 (rs900400)				ADCY5 (rs9883204)			
	TT (n = 1258)	TC (n = 679)	CC (n = 96)	P value	TT (n = 87)	CT (n = 610)	CC (n = 1339)	P value
Age (years)	28.4 (1.12)	28.4 (1.08)	28.2 (1.06)	0.59	28.3 (1.13)	28.4 (1.12)	28.3 (1.09)	0.49
Gender (% female)	582 (46.26)	360 (53.02)	39 (40.63)	0.86	43 (49.43)	301 (49.34)	644 (48.10)	0.006
Birth Weight (kg)	2.79 (0.47)	2.80 (0.45)	2.80 (0.53)	0.87	2.77 (0.55)	2.80 (0.46)	2.78 (0.46)	0.54
Adult Weight (kg)	53.2 (11.58)	53.3 (11.60)	55.5 (12.18)	0.13	52.4 (11.97)	53.2 (11.44)	53.5 (11.65)	0.80
BMI (kg/m^2^)	20.6 (3.79)	20.8 (3.81)	21.0 (3.94)	0.42	20.3 (4.16)	20.7 (3.80)	20.7 (3.76)	0.64
Waist circumference (cm)	74 (10.5)	74 (10.6)	76 (10.4)	0.21	73 (9.62)	74 (10.63)	74 (10.48)	0.66
Hip circumference (cm)	88 (8.2)	88 (8.1)	88 (8.5)	0.39	87 (8.98)	88 (8.04)	88(8.15)	0.78
Systolic BP (mm Hg)	107 (12.2)	106 (11.9)	109 (13.6)	0.39	106 (11.9)	107 (11.9)	107 (12.3)	0.44
Diastolic BP (mm Hg)	72 (8.89)	73 (9.05)	73 (8.53)	0.44	71 (8.32)	72 (8.96)	73 (8.82)	0.11
*Body fat %	23.3 (9.63)	24.5 (9.54)	23.0 (9.76)	0.52	23.0 (10.10)	23.7 (9.83)	23.7 (9.51)	0.44
Skin fold thickness: Triceps (mm)	10.3 (6.6,16.5)	11 (7.1,17.5)	10.5 (6.1,15.1)	0.07	8.7 (5.9,17.4)	10.9 (7,17.2)	10.5 (6.8,16.5)	0.22
Skin fold thickness: Biceps (mm)	4.8 (3.3,7.6)	5.3 (3.4,8)	4.4 (3.4,7.3)	0.09	4.5 (3,8.1)	5 (3.5,8)	5 (3.4,7.7)	0.57
Skin fold thickness: Subscapular (mm)	16.7 (10.7,27.3)	17.1 (11.4,28.6)	15.3 (10.1,29.4)	0.26	16.8 (9.8,26.8)	16.9 (11,28.3)	16.9 810.9,28)	0.62
Skin fold thickness: Abdomen (mm)	18.5 (9.6,32.3)	19.9 (11.1,33.5)	22.7 (10, 33.5)	0.15	17.7 (8.4,29.3)	18.8 (10.1,32.6)	19.5 (10.1,33.4)	0.35
Total Cholesterol (mmol/l)	4.0 (0.89)	4.0 (0.83)	4.1 (0.89)	0.55	3.7 (0.76)	4.0 (0.87)	3.9 (0.87)	0.033
Triglycerides (mmol/l)	1.1 (0.70)	1.1 (0.60)	1.1 (0.78)	0.97	1.1 (0.68)	1.0 (0.63)	1.1 (0.69)	0.33
HDL-C (mmol/l)	1.0 (0.24)	1.0 (0.24)	1.1 (0.24)	0.35	1.0 (0.20)	1.0 (0.24)	1.0 (0.24)	0.33
LDL-C (mmol/l)	2.4 (0.74)	2.5 (0.72)	2. 5(0.75)	0.53	2.3 (0.64)	2.5 (0.76)	2.4 (0.72)	0.017
			

Data represented as Mean (SD) for all traits. Skin fold thickness is presented as median ( Interquartile range).

Comparing birth weights in extreme homozygotes (CC) for both of the ‘birth weight-lowering’ variants, no statistically significant difference between carriers (2,772±535 g, n = 62) and non-carriers (2,782±552 g, n = 53, *p* = 0.10) was observed.

We then compared the birth weights for participants with normoglycemia, IFG, IGT and manifest type 2 diabetes (T2DM) in the entire cohort. The mean birth weight among people with normoglycemia was 2,795±460 g (n = 1,694), with IFG (n = 83) 2,754±505 g, with IGT (n = 319) was 2,763±496 g and 2,715±536 g in the diabetes group (n = 55). The difference appeared largest between the normoglycemic group and the groups with manifest type 2 diabetes, but this was not statistically significant (*p* = 0.24).

There was no significant relationship between either of the genotypes and Z-scores of anthropometry measured across various time periods at birth, infancy, childhood, adolescence or adulthood (Table S1). Neither of the SNPs in additive model showed significant association with any of the anthropometric measures of obesity in adulthood. (Table 1), and this signal was not picked up by either the BMI or waist circumference measurements. The additive model of *ADCY5* variant was associated with raised 2-hour glucose postprandially (*p* = 0.010) (Table 2).

**Table 2:** Association of glycemic traits with rs9883204 (*ADCY5*) additive model.

	CC		CT		TT		P value†	Effect (95%CI)	P value‡
	n	Mean (SD)	n	Mean (SD)	n	Mean (SD)			
Fasting Glucose (mmol/l) a	1339	5.28 (0.42)	610	5.34 (0.46)	87	5.38 (0.51)	0.13	0.041 (0.004, 0.078)	0.027
2hour Glucose (mmol/l) a	1339	6.34 (1.41)	610	6.19 (1.35)	87	6.40 (1.49)	0.010	0.127 (0.021, 0.233)	0.019
Fasting Insulin (pmol/l) a	1076	36.81 (19.45,61.12)	497	37.50 (21.53,61.12)	75	37.85 (20.48,63.209	0.10	−0.008 (−0.073, 0.057)	0.81
2 hour Insulin (pmol/l) a	1265	168.07 (100–295.16)	584	153.14 (86.81,255.58)	85	149.32 (82.64,268.07)	0.24	−0.058 (−0.126, 0.010)	0.010
Insulinogenic Index b, c	992	3.11 (1.39,5.89)	471	2.66 (1.32,5.82)	74	2.44 (1.15,5.16)	0.23	−0.106 (−0.211, −0.0006)	0.050
AUC glucose b, ^d^	1337	7.12 (6.35,7.91)	610	6.33 (5.74,8.06)	87	7.20 (6.39,8.16)	0.15	0.005 (−0.0005, 0.010)	0.08
AUC Insulin b, ^d^	1333	1.22 (0.79,2.01)	608	1.27 (0.72,2.09)	87	1.19 (0.69,2.02)	0.33	−0.061 (−0.124, 0.002)	0.057
HOMA IR b	1336	1.12 (0.50,1.94)	610	1.02 (0.42,1.86)	87	0.99 (0.37,1.88)	0.63	−0.059 (−0.016, 0.043)	0.26
HOMA B b	1336	47.20 (24.91,93.09)	610	48.15 (18.79,85.13)	87	44.55 (17.16,86.09)	0.32	−0.089 (−0.189, 0.012)	0.09

Data represented as ^a^mean (SD) and ^b^median (Inter-quartile range).

† P values adjusted for gender.

‡ P values obtained by regression models adjusted for age, gender, consanguinity and BMI.

C Insulinogenic index  =  (Insulin _30_-fasting Insulin) / ( glucose_30_-fasting glucose).

Regression analysis showed that the ‘birth weight-lowering’ variant of *ADCY5,* was significantly and positively associated with increase in 2-hour glucose, fasting glucose, reduced insulinogenic index and reduced 2-hour insulin (Table 2). Since the rise in 2-hour glucose is dependent on the rate of peripheral glucose disposal, we presumed that fasting glucose may confound this association. However, we found that the association of *ADCY5* variant with 2-hour glucose remained significant even after adjusting for fasting glucose (Logged β 0.102, *p* = 0.053). The ‘birth weight-lowering’ variants were not associated with calculated index of insulin resistance (HOMA-IR). No impairment of β-cell function was observed for variants at the *CCNL1* locus (Table 3).

**Table 3:** Association of glycemic traits with rs900400 (*CCNL1*) additive model.

	TT		TC		CC		P value†	Effect (95%CI)	P value‡
	n	Mean (SD)	n	Mean (SD)	n	Mean (SD)			
Fasting Glucose (mmol/l)^ a^	1258	5.35 (0.48)	679	5.37 (0.51)	96	5.45 (0.56)	0.18	0.005 (−0.002, 0.012)	0.15
2hour Glucose (mmol/l) a	1258	6.31 (1.48)	679	6.40 (1.40)	96	6.24 (1.32)	0.57	0.004 (−0.010, 0.108)	0.94
Fasting Insulin (pmol/l) a	1009	37.5 (21.5,62.5)	554	37.5 (19.5,61.1)	87	40.9 (21.5,65.3)	0.82	−0.00006 (−0.062, 0.063)	0.99
2 hour Insulin (pmol/l) a	1196	146.5 (82.7,261.1)	644	158.4 (90.3,267.7)	91	150.7 (74.3–286.8)	0.35	0.028 (−0.039, 0.095)	0.41
Insulinogenic Index b, c	941	2.43 (1.23,5.22)	516	2.62 (1.10,5.63)	81	2.58 (1.03,5.31)	0.92	−0.023 (−0.127, 0.112)	0.67
AUC glucose b, ^d^	1257	7.19 (6.41,8.12)	678	7.23 (6.38,8.15)	96	7.07 (6.23,7.91)	0.51	0.00005 (−0.005, 0.005)	0.99
AUC Insulin b ^d^	1249	1.19 (0.70,1.98)	679	1.23 (0.71,2.12)	96	1.27 (0.78,1.97)	0.48	0.018 (−0.043, 0.080)	0.56
HOMA IR b	1254	1.01 (0.39,1.84)	679	0.97 (0.38,1.87)	96	1.29 (0.62,2.08)	0.11	0.073 (−0.026, 0.171)	0.15
HOMA B b	1254	45.76 (18.05,86.62)	679	45.99 (18.04,83.44)	96	59.53 (27.22,85.35)	0.17	0.059 (−0.039, 0.158)	0.24

Data represented as ^a^mean (SD) and ^b^median (Inter-quartile range).

† P values adjusted for gender

‡ P values obtained by regression models adjusted for age, gender, consanguinity and BMI.

C Insulinogenic index  =  (Insulin _30_-fasting Insulin) / ( glucose_30_-fasting glucose)

To further analyze the impact on postprandial glycemia in the homozygous carriers of the *ADCY5* variant, we calculated the frequency of having plasma glucose above an arbitrary cut-off of 9 and 11 mmol/L, respectively. The lower level was chosen to enhance the capacity to detect even lower glycemic disturbances in these apparently healthy and young adults. At the 9 mmol/L cut off, the odds ratio (OR) and 95% confidence was 1.64 (1.08–2.48) *p* = 0.016, whereas there was no significant effect at the 11 mmol/L cut off (OR 1.07, 95%CI 0.56–2.04, *p = *0.84).

## Discussion

Genetic variants associated with a birth weight-lowering effect in other populations, near *CCNL1* or in *ADCY5* locus are not associated with birth weight in this Indian cohort, but the ‘birth weight-lowering’ variant of *ADCY5* was associated with raised glucose and reduced early phase insulin secretion.

The Indian birth weight is generally about 0.8 kg less compared to the West [21], and the mean birth weight in our cohort was 2.8 kg. Not only was there any modulation between genotypes at this lower level, the gene frequencies of the ‘birth weight-lowering’ variants in *ADCY5* and near *CCNL* were also the same (*ADCY5*) or lower (*CCNL*) than in the original report in Europeans [1]. The absence of an association between ‘birth weight-lowering’ genetic variants and birth weight is therefore likely to be attributed to strong environmental influences which dominate over the genetic effects. This would be supported by the observations of slightly higher birth weights in Indian immigrants in the West compared to the native Indian babies [22], [23]. The existence of other genetic variants influencing birth weight in the Indian setting is possible, but rather unlikely considering the considerable genetic diversity in India [24] contrasted with the uniformity in the low birth weight phenotype.

The *ADCY5* variant has been associated with increased plasma fasting glucose at a genome-wide level [15] and it appears that, type 2 diabetes genes in general are reproduced in Indians [25]. In the current study, rs9883204 variant of *ADCY5* was associated with raised fasting and 2-hour glucose concentrations following an oral glucose tolerance test performed in early adulthood. Although this finding would be in general agreement with the fetal-insulin hypothesis, it is paradoxical compared to a recent study in a large Danish cohort as another ‘birth weight-lowering’ (rs11708067) variant was associated with reduced adult insulin resistance [16]. The *ADCY5* rs11708067 and rs9883204 are in close linkage disequilibrium in the Hapmap CEPH (Utah residents with ancestry from northern and western Europe) population data (D' 0.93, r2 0.72, Hapmap data rel 27 - http://hapmap.ncbi.nlm.nih.gov). rs9883204 is not represented in the Hapmap Indian population (GIH - Gujarathi Indian population), therefore LD cannot be calculated directly within Hapmap. However, a further SNP, rs17361324, which is a proxy for rs9883204 in the CEPH Hapmap data is also a proxy for rs11708067 in the GIH Hapmap data. This may suggest that, the high LD between these two SNPs in the CEPH data is also present in the Indian population and the difference does not appear to be explained by the genetic architecture and will need confirmation in future studies. Additionally, the low birth weight and T2DM risk allele of *ADCY5* variant was associated with reduced early insulin response measured by insulinogenic index, consistent with original report by Freathy et al., supportive of a possible role in influencing insulin secretion [1]. It is proposed that *ADCY5* risk allele may operate by different mechanisms by which they influence birth weight and T2DM risk susceptibility [16], the later probably may be through an effect on insulin secretion rather than insulin resistance. It is worth mentioning that for glucose and insulin, the association detailed in this cohort shows borderline significance and type 2 error cannot be excluded.

The loss of heterozygosity of rs9883204 in our population is probably related to the high endogamy which is commonly seen among Indians. It is generally assumed that an association detected by a well-powered GWAS is in LD with the functional variant. The strong signals in GWAS are related to the functional magnitude of the effect and therefore, we assume this holds good in our population despite a considerable degree of consanguinity.

Although, we have studied, the genetic variants associated with birth weight in one of the largest and homogenous birth cohorts from India, our study has limitations. Our study was adequately powered for *CCNL1*, but less strong for *ADCY5* to explain the variance observed with these two SNPs. The CV for birth weight in our study was comparable to originally described and this further solidifies our power to detect an association. Also the lower mean birth weight, observed in this population, might potentially be associated with a reduced overall variance in the dependent variable, i.e., reduce the effect of the functional variant on birth weight, which could possibly contribute to the lowered power in our study. Although it is evident that a multiple comparison correction would abolish the modest associations with glycemic traits, we believe that the association is biologically relevant and that loss of statistical significance by multiple testing does not necessarily disprove a true association owing to the homogenous population studied within a small geographical region and the similar allele frequencies reported among Caucasians [1]. We did not have the maternal genotype to assess the effect of maternal genetic variants on birth weight due to non-availability of blood sample from the mothers. The low number of diabetes cases in this still young cohort did not allow for observing a possible link between diabetes incidence and birth weight or genetic links between them.

In conclusion, the ‘birth weight-lowering’ variants in *ADCY5* and near *CCNL1* showing strong associations with birth weight in European cohorts appear to have little or no effect in the Indian setting. However, the ‘birth weight-lowering’ variant in *ADCY5* was associated with modest glucose intolerance in early adulthood which reinforces the argument for a genetic link between in utero growth and adult type 2 diabetes.

## Supporting Information

Table S1 Association between rs900400 and rs9883204 and Z-scores of anthropometric traits from birth to adulthood.
(DOC)
