# Supplementary material for: Absence of Birth-Weight Lowering Effect of ADCY5 and Near CCNL, but Association of Impaired Glucose-Insulin Homeostasis with ADCY5 in Asian Indians
Source: PLoS One. 2011 Jun 21;6(6):e21331. doi: 10.1371/journal.pone.0021331 (PMC3119677; doi:10.1371/journal.pone.0021331)
Supplement: Table S1 — Association between rs900400 and rs9883204 and Z-scores of anthropometric traits from birth to adulthood. (DOC) [file pone.0021331.s001.doc]

| Supplement Table 1. Association between rs900400 and rs9883204 and Z-scores of anthropometric traits from birth to adulthood | | | | | | | | | | | | |
| --- | --- | --- | --- | --- | --- | --- | --- | --- | --- | --- | --- | --- |
| Z Scores | CCNL 1 ( rs900400) | | | | | | ADCY5 (rs9883204) | | | | | |
|  | TT (n=1296) | TC (n=694) | CC (n=97) | P value† | Beta (95%CI) | P value‡ | TT (n=90) | CT (n=621) | CC (n=1380) | P value† | Beta ( SE) | P value‡ |
| Birth weight | 2.79 (4.72) | 2.80 (4.48) | 2.80 (5.25) | 0.94 | -8.27 (-46.66, 30.12) | 0.67 | 2.77 (5.45) | 2.81 (4.64) | 2.78 (4.63) | 0.57 | -15.43 (-54.05, 23.19) | 0.43 |
| Z- birth weight | -0.0008  (-0.649,0.583) | 0.005  (-0.639,0.549) | -0.156  (-0.644,0.752) | 0.86 | -0.018 (0.010, 0.645) | 0.68 | -0.156  (-0.684, 0.549) | 0.002  (-0.684, 0.549) | 0.002  (-0.649, 0.549) | 0.55 | -0.033 (-0.116, 0.050) | 0.44 |
| Z- birth length | 0.013  (-0.623,0.595) | -0.059  (-0.623,0.594) | 0.062  (-0.623,0.540) | 0.77 | 0.006 (-0.074, 0.086) | 0.88 | -0.119  (-0.701, 0.594) | 0.013  (-0.641, 0.594) | 0.013  (-0.584, 0.540) | 0.96 | -0.012 (-0.092, 0.069) | 0.77 |
| Z- head circumference | 0.101  (-0.556, 0.621) | 0.101  (-0.542, 0.692) | 0.103  (-0.054, 0.751) | 0.50 | 0.030 (-0.051, 0.110) | 0.47 | 0.101  (-0.542, 0.627) | 0.101  (-0.556, 0.751) | 0.101  (-0.542, 0.430) | 0.79 | -0.015 (-0.096, 0.065) | 0.71 |
| Z-birth BMI ^a^ | -0.597  (-0.497,0.583) | -0.059  (-0.0542,0.692) | -0.034  (-0.0683,0.600) | 0.86 | -0.021 (-0.103, 0.061) | 0.62 | 0.101  (-0.542,0.627) | -0.073  (-0.590,0.662) | 0.101  (-0.0542,0.430) | 0.35 | -0.029 (-0.112, 0.053) | 0.49 |
| Z- Infancy weight | -0.00004  (-0.649, 0.592) | -0.022  ( -0.724, 0.066) | 0.091  (-0.442, 0.758) | 0.37 | 0.017 (-0.075, 0.109) | 0.71 | 0.044  (-0.503,0.482) | 0.058  (-0.625, 0.614) | -0.0005  (-0.672, 0.634) | 0.72 | -0.046 (-0.140, 0.047) | 0.33 |
| Z -Infancy height | 0.240  ( -0.643, 0.654) | 0.102  (-0.552, 0.695) | 0.158  (-0.579, 0.728) | 0.40 | 0.029 (-0.064, 0.122) | 0.54 | -0.075  (-0.774, 0.676) | 0.065  (-0.594, 0.628) | 0.065  (-0.061, 0.663) | 0.62 | -0.006 (-0.100, 0.088) | 0.90 |
| Z-Infancy head circumference | 0.059  (-0.636, 0.652) | 0.059  (-0.596, 0.755) | 0.059  (-0.636, 0.838) | 0.71 | 0.007 (-0.086, 0.099) | 0.89 | 0.063  (-0.325,0.652) | -0.076  (-0.636, 0.652) | 0.059  (-0.636, 0.754) | 0.44 | 0.012 (-0.083, 0.106) | 0.81 |
| Z- Infancy BMI | -0.004  (-0.651, 0.634) | -0.023  (-0.712, -0.475) | -0.018  (-0.446, 0.533) | 0.24 | -0.046 (-0.142, 0.051) | 0.35 | -0.115  (0.556, 0.733) | -0.040  (-0.671, 0.585) | -0.007  (-0.671, 0.573) | 0.72 | -0.027 (-0.124, 0.071) | 0.59 |
| Z- Childhood weight | -0.059  (-0.657, 0.486) | -0.041  ( -0.607, 0.585) | 0.117  (-0.713, 0.767) | 0.45 | -0.011 (-0.092, 0.071) | 0.79 | -0.167  (-0.681, 0.555) | 0.039  (-0.628, 0.522) | -0.059  (-0.653, 0.522) | 0.71 | -0.050 (-0.132, 0.032) | 0.23 |
| Z- Childhood height | -0.016  (-0.639, 0.625) | 0.065  (-0.599, 0.664) | 0.143  (-0.325, 0.838) | 0.25 | 0.022 (-0.062, 0.106) | 0.60 | 0.051  (-0.495, 0.813) | 0.037  (-0.646, 0.664) | -0.001  (-0.602,0.641) | 0.50 | -0.043 (-0.128, 0.041) | 0.32 |
| Z- Childhood head circumference | -0.055  (-0.645, 0.588) | 0.037  (-0.540, 0.066) | 0.052  (-0.580, 0.761) | 0.21 | 0.037 (-0.047, 0.123) | 0.39 | 0.041  (-0.645, 0.609) | -0.014  (-0.624, 0.559) | 0.001  (-0.602, 0.641) | 0.95 | -0.003 (-0.090, 0.084) | 0.94 |
| Z- Childhood BMI | -0.101  (-0.609, 0.608) | -0.020  (-0.619, 0.551) | -0.012  (-0.456, 0.424) | 0.98 | -0.037 (-0.116, 0.042) | 0.35 | -0.161  (-0.757, 0.726) | -0.093  (-0.590, 0.602) | -0.076  (-0.616, 0.552) | 0.97 | -0.024 (-0.104, 0.057) | 0.56 |
| Z- Adolescent weight | -0.115  (-0.719, 0.534) | -0.037  (-0.063, 0.113) | 0.142  (-0.748, 0.592) | 0.25 | 0.023 (-0.069, 0.115) | 0.62 | -0.237  (-0.733,0.463) | 0.013  (-0.685, 0.605) | -0.097  (-0.735, 0.545) | 0.41 | -0.022 (0.116, 0.072) | 0.65 |
| Z- Adolescent height | -0.044  (-0.715, 0.655) | -0.032  (-0.645, 0.705) | 0.162  (-0.607, 0.721) | 0.33 | 0.008 (-0.082, 0.990) | 0.86 | -0.177  (-0.738, 0.620) | 0.010  (0.687, 0.669) | -0.054  (-0.689, 0.672) | 0.86 | 0.018 (-0.074, 0.110) | 0.70 |
| Z- Adolescent BMI | -0.066  (-0.654, 0.641) | -0.066  (-0.654, 0.641) | 0.641  (-0.599, 0.664) | 0.59 | 0.025 (-0.068, 0.118) | 0.60 | 0.031  (-0.911, 0.532) | -0.001  (-0.536, 0.662) | -0.106  (-0.714, 0.604) | 0.31 | -0.038 (-0.133, 0.057) | 0.43 |
| Z- Adult weight | -0.221  (-0.776, 0.568) | -0.134  (-0.717, 0.665) | -0.115  (-0.664, 0.815) | 0.13 | 0.068 (-0.014, 0.150) | 0.11 | -0.256  (-0.930, 0.453) | -0.209  (-0.729, 0.642) | -0.221  (-0.753, 0.594) | 0.82 | -0.008 (-0.091, 0.074) | 0.85 |
| Z- Adult height | -0.006  (-0.698, 0.633) | 0.097  (-0.628, 0.682) | 0.167  (-0.430, 0.780) | 0.11 | 0.051 (-0.030, 0.132) | 0.21 | 0.800  (-0.802, 0.712) | 0.001  (-0.628, 0.606) | -0.031  (-0.696, 0.667) | 0.80 | -0.024 (-0.105, 0.057) | 0.57 |
| Z- Adult BMI | -0.226  (-0.785, 0.578) | -0.185  (-0.781, 0.667) | -0.103  (-0.723, 0.793) | 0.40 | 0.054 (-0.028, 0.137) | 0.20 | -0.264  (-0.899, 0.491) | -0.232  (-0.756, 0.646) | -0.195  (-0.770, 0.636) | 0.56 | 0.006 (-0.077, 0.089) | 0.88 |
| Data presented as Median (IQR) and ^a^mean (SD). †, ‡ All variables adjusted for gestational age, consanguinity and gender. | | | | | | | | | | | | |
